# Prevalence of hookworm infection and its proportion among pregnant women with intestinal helminth infection in Ethiopia: A systematic review and meta-analysis

**DOI:** 10.1371/journal.pntd.0014376

**Published:** 2026-06-10

**Authors:** Mengistie Kassahun Tariku, Gezahegn Eshetu Mekuriya, Habtamu Wagnew Abuhay, Lidetu Demoze, Gedefaw Abeje, Mekuriaw Nibret Aweke, Habtamu Abebe Getahun, Samuel Teferi Chanie, Gelila Yitageasu, Gebrie Getu Alemu, Asebe Hagos

**Affiliations:** 1 Department of Public Health, College of Health Science, Debre Markos University, Debre Markos, Ethiopia; 2 Department of Epidemiology and Biostatistics, College of Medicine and Health Science, Bahir Dar University, Bahir Dar, Ethiopia; 3 EpiGen Ethiopia, Bahir Dar University, Bahir Dar, Ethiopia; 4 Department of Human Nutrition and Dietetics, Institute of Public Health, College of Medicine and Health Sciences, University of Gondar, Gondar, Ethiopia; 5 Department of Epidemiology and Biostatistics, Institute of Public Health, College of Medicine and Health Sciences, University of Gondar, Gondar, Ethiopia; 6 School of Population Health, Faculty of Health Sciences, Curtin University, Bentley, Western Australia, Australia; 7 Department of Environmental and Occupational Health and Safety, Institute of Public Health, College of Medicine and Health Sciences, University of Gondar, Gondar, Ethiopia; 8 Department of Nursing, Debre Tabor Health Science College, Gondar, Debre Tabor, Ethiopia; 9 Department of Human Nutrition, Institute of Public Health, College of Medicine and Health Sciences, University of Gondar, Gondar, Ethiopia; 10 Department of Physiotherapy, School of Medicine, College of Medicine and Health Sciences, University of Gondar, Gondar, Ethiopia; 11 Department of Health Systems and Policy, Institute of Public Health, College of Medicine and Health Sciences, University of Gondar, Gondar, Ethiopia; The University of Melbourne, AUSTRALIA

## Abstract

**Background:**

Hookworms are the most common soil-transmitted helminths in tropical and subtropical regions. Although infection with hookworms is a principal cause of maternal anemia, there is limited consolidated evidence on pooled prevalence and its proportion among intestinal helminths infections in pregnant women in Ethiopia. This study aimed to estimate the pooled prevalence of infection with hookworms and its proportion among intestinal helminths in pregnant women in Ethiopia.

**Methods:**

A systematic review and meta-analysis were conducted following the PRISMA guidelines. Articles published between 2010 and 2024 were retrieved from databases including PubMed, Google Scholar, ScienceDirect, and African Journals Online using relevant search terms related to Ethiopia. Data extraction was performed using a standardized Excel spreadsheet after assessing the quality of the studies with the Newcastle-Ottawa Scale. Data were analyzed using STATA version 17. A random-effects model using the DerSimonian–Laird method was employed to account for heterogeneity, which was assessed using the I² statistic and the *p*-value of Cochrane’s Q test. Publication bias was evaluated using funnel plots, Begg’s, and Egger’s tests. Results were presented using forest plots with 95% confidence intervals (CIs).

**Results:**

A total of 33 studies, including 13367 pregnant women were analysed. Among these 4161 women infected with intestinal Helminthiases, including 1853 cases of infection with hookworms. The pooled prevalence of infection with hookworms among pregnant women in Ethiopia was 12.21% (95% CI: 9.46%–14.95%), while the pooled proportion of hookworms among intestinal helminths was 44.0% (95% CI: 33.24%–55.75%). Regionally, prevalence was highest in Amhara (13.11%, 95% CI: 9.17%–17.05%) and the lowest in Gambella (4.44%, 95% CI: 2.32%–6.57%). The proportion was highest in Amhara (67.86%, 95% CI: 57.87%–77.84%) and lowest in Harerge (16.67%, 95% CI: 9.21%–24.12%). By diagnostic method, the concentration technique yielded the highest prevalence (16.11%, 95% CI: 9.31%–22.90%), while combined methods showed the lowest (9.48%, 95% CI: 6.14%–12.83%).

**Conclusion:**

Infection with hookworms remains a public health concern among pregnant women in Ethiopia (pooled prevalence: 12.21%), accounting for 44.0% of intestinal helminths. Marked regional variation exists, highest in Amhara and lowest in Gambella. Prevalence estimates varied by diagnostic method, with higher values from concentration techniques. Targeted interventions, improved sanitation, deworming, and standardized diagnostics are needed.

## Author summary

Hookworms are common soil-transmitted helminthic disease in tropical and subtropical regions, including Ethiopia. While low-intensity infections are often asymptomatic, moderate-to-heavy infections can contribute substantially to anemia and adverse maternal health outcomes, particularly among pregnant women. Despite its public health importance, national-level evidence on the overall burden of infection with hookworms in this population has been limited and fragmented. In this study, we systematically reviewed and analyzed data from multiple studies conducted across Ethiopia to estimate both the overall prevalence of infection with hookworms among pregnant women and its proportion among intestinal helminth infections.

Our findings showed that about one in eight pregnant women in Ethiopia were infected with hookworms, and nearly half of all intestinal helminths infections in this group were due to hookworms. We also found significant regional differences, with higher prevalence in some areas than others, and variations depending on the diagnostic methods used.

These results highlight that infection with hookworms remains a significant public health concern for pregnant women in Ethiopia. Strengthening prevention strategies such as improving sanitation, promoting the use of footwear, and expanding deworming programs will be essential to reduce infection and improve maternal health outcomes.

## Introduction

Hookworm infection, caused by several species of hookworms, is the second most common soil-transmitted helminth infection globally, following infection caused by *Ascaris lumbricoides* [1,2]. It is primarily transmitted through contact with contaminated soil, especially by walking barefoot, and is most prevalent in tropical and subtropical regions with warm, moist climates conducive to larval survival [2]. Globally, an estimated 576–740 million people are infected with hookworms [1], with approximately 688 million women of reproductive age living in endemic areas [3].

Sub-Saharan Africa bears a significant burden of infection with hookworms. Each year, an estimated 37.7 million women of reproductive age and 6.9 million pregnant women in the region are infected [4].

Infection with Hookworms is a major contributor to morbidity, accounting for over 4 million disability-adjusted life years (DALYs) globally. Its economic impact is substantial, with productivity losses estimated to range from $7.5 billion to $138.9 billion annually [5].

In pregnancy, infection with hookworms is associated with serious maternal and fetal complications, including anemia, malnutrition, heart failure, ascites, low birth weight, neonatal prematurity, and even prenatal mortality [6]. It also impairs fetal growth and cognitive development [7]. Risk factors for infection with hookworms include low socioeconomic status, poor sanitation, walking barefoot, and inadequate personal hygiene [8].

Despite global recommendations, preventive chemotherapy coverage for pregnant women remains low, reaching only 10–20% [9]. As part of the 2030 global targets for STH control, integrating deworming into routine antenatal care is recognized as a key strategy to reduce maternal anemia and worm burden [10]. A single dose of antihelminthics administered during the second trimester has been shown to be safe and effective [11]. Establishing an efficient STH control program in pregnant women is one of six 2030 global targets for STH [12].

While several studies have addressed the prevalence and effects of intestinal helminths during pregnancy [13,14], evidence specific to the epidemiology of infection with hookworms among pregnant women in Ethiopia remains limited and fragmented [15,16]. A systematic synthesis of existing data is essential for informing policy and tailoring interventions [17].

Therefore, this systematic review and meta-analysis aimed to estimate the pooled prevalence of infection with hookworms and its proportion among pregnant women with intestinal helmenthiases in Ethiopia and to summarize key epidemiological characteristics that can inform targeted public health interventions [18].

## Methods

### Study design and setting

A systematic review and meta-analysis were conducted using computerized database searches focused on Ethiopia, a country located in the Horn of Africa. Ethiopia is bordered by Eritrea to the north, Sudan to the northwest, South Sudan to the west, Kenya to the southwest, and Somalia to the southeast and east. The country’s capital, Addis Ababa, is situated in the central part of the nation. According to Worldometer, as of Wednesday, July 2, 2025, Ethiopia had an estimated total population of 135,482,620 and a fertility rate of 4.3. This population accounts for ~1.47% of the global population, ranking Ethiopia 12th in the world by population size [19].

### Search strategy

Before conducting the electronic database search, a search strategy was developed using the following common keywords: “prevalence”, “magnitude”, or “proportion”; “hookworm infection”, “soil-transmitted helminths”, “intestinal helminths”, or “intestinal parasites”; “pregnant women” or “resource-limited setting”; and “Ethiopia”. These terms were combined using the Boolean operators AND and OR. A comprehensive literature search was then carried out across multiple databases, including Google Scholar, PubMed, ScienceDirect, and African Journals Online, to identify relevant studies. The results of the systematic review and meta-analysis were reported in accordance with the Preferred Reporting Items for Systematic Reviews and Meta-Analyses (PRISMA) guidelines [20] (S1 PRISMA Checklist). We have registered in Prospero with the registration number of CRD420251084892.

### Eligibility criteria

#### Inclusion criteria.

Studies were included in this systematic review and meta-analysis if they met predefined criteria. Eligible studies were those conducted among pregnant women in Ethiopia that reported data on infection with hookworms. To ensure accurate estimation of prevalence, only observational studies with appropriate population denominators were considered, including cross-sectional studies and baseline data from cohort studies. Studies were required to report both the total number of pregnant women screened and the number of hookworm infections, allowing for the calculation of prevalence. Additionally, only articles published in peer-reviewed journals between 2010 and 2024 and written in English were included.

No restrictions were applied regarding participants’ age, geographic location within Ethiopia, or healthcare setting, provided that the study population consisted of pregnant women. Furthermore, a minimum of two eligible studies was required for inclusion in the meta-analysis to allow for the calculation of a pooled prevalence estimate.

#### Hookworms diagnosis.

In Ethiopia, hookworm diagnosis is predominantly based on stool microscopy techniques, including direct wet-mount, Kato–Katz, and formol-ether concentration methods. Combined methods refer to the use of two or more diagnostic techniques within the same study to detect hookworm infections, such as direct wet-mount microscopy together with concentration techniques (e.g., formal-ether concentration or Kato–Katz methods). These approaches are commonly employed in epidemiological studies to improve sensitivity and detection of low-intensity infections.

#### Exclusion criteria.

Studies were excluded if the full text could not be retrieved after reasonable efforts or if they did not provide sufficient data to determine the number of infection hookworms and the total number of pregnant women screened. Articles that did not clearly report outcomes related to infection with hookworms among pregnant women were also excluded. Study designs that lacked appropriate denominators for estimating prevalence, such as case-control studies without baseline data, were not considered. In addition, non-primary research articles—including review articles, systematic reviews, meta-analyses, editorials, letters, and conference abstracts—were excluded, as were duplicate publications.

### Outcome variable

This systematic review and meta-analysis estimated the prevalence of infection with hookworms among pregnant women. The primary outcome was calculated by dividing the number of pregnant women diagnosed with hookworm infections by the total number of pregnant women screened in each study. In addition, to allow comparison with previous studies reporting parasite distribution, a secondary outcome was calculated as the proportion of infection with hookworms among all intestinal helminths infections, obtained by dividing the number of hookworms cases by the total number of intestinal helminths infections and multiplying by 100. Both measures were analyzed and reported to provide a comprehensive assessment of the burden and relative contribution of infection with hookworms among intestinal helminths.

### Data extraction

Prior to data extraction, a standardized data extraction spreadsheet was developed using Microsoft Excel (S1 File and S1 Data). The tool was independently reviewed by two authors, and a consensus was reached on its final form. The data extraction form included the following variables: author’s name, publication year, study setting (institution-based or community based), study area/region, study design, study period, diagnostic method, sample size, number of hookworm infections, and prevalence rate. All published articles from 2010 to 2024 were screened, and relevant data were extracted independently by two authors between January 1, 2023, and December 30, 2024.

### Quality appraisal

The quality of the included observational studies was assessed using the Newcastle-Ottawa Scale [21] quality appraisal tool [22]. This tool comprises three sections: the first section, with a maximum of five stars, evaluates the representativeness of the sample, sample size, non-response rate, and ascertainment of exposure; the second section, with up to two stars, assesses the comparability of study outcomes or exposures; and the third section, with up to three stars, evaluates outcome assessment and the appropriateness of statistical analysis. All included studies were independently appraised by two authors. Discrepancies in scoring were resolved through discussion before determining the final quality score. Studies scoring ≥6 out of 10 were considered high quality and were included in the meta-analysis. Notably, all eligible studies met this quality threshold, and none were excluded at this stage (S1 File).

### Data processing and analysis

Relevant data were extracted using an Excel spreadsheet and then exported to STATA version 17 for meta-analysis. The systematic review primarily focused on summarizing the study area/region, study settings (community- or institution-based), study design, diagnostic methods used for detecting infection with hookworm, sample size, and the reported prevalence and its proportion of infection with hookworms among pregnant women. The meta-analysis aimed to compute the overall pooled prevalence and its proportion of infection with hookworms, as well as to perform subgroup analyses.

To estimate the pooled prevalence and proportion, the 95% CI and standard error of each study were used. Subgroup analyses were conducted based on study area/region, study setting, study design, and diagnostic method. A random-effects model using the DerSimonian–Laird method was applied to account for heterogeneity among the included studies. Study weights were assigned using the inverse-variance method, in which studies with greater precision contributed proportionally more to the pooled estimate. Heterogeneity among the included studies was assessed using the I² statistic and the p-value of the Cochrane Q test. An I² value greater than 75% was considered indicative of substantial heterogeneity. To explore potential sources of heterogeneity, subgroup analyses were conducted based on study area or region, study setting (community based Studies: conducted in the general population or specific geographic communities outside institutional settings or institution-based: studies conducted in healthcare (e.g., hospitals, antenatal clinics) where participants are recruited from institutional settings), study design, and diagnostic method used for detecting infection with hookworms.

To evaluate potential publication bias, visual inspection of the funnel plot was conducted, along with Begg’s and Egger’s tests [23]. Trim and fill analysis was conducted to adjust publication bias. Finally, the results were presented using a forest plot showing the pooled prevalence and its proportion with a 95% CI.

## Results‌‌

**Fig 1:**
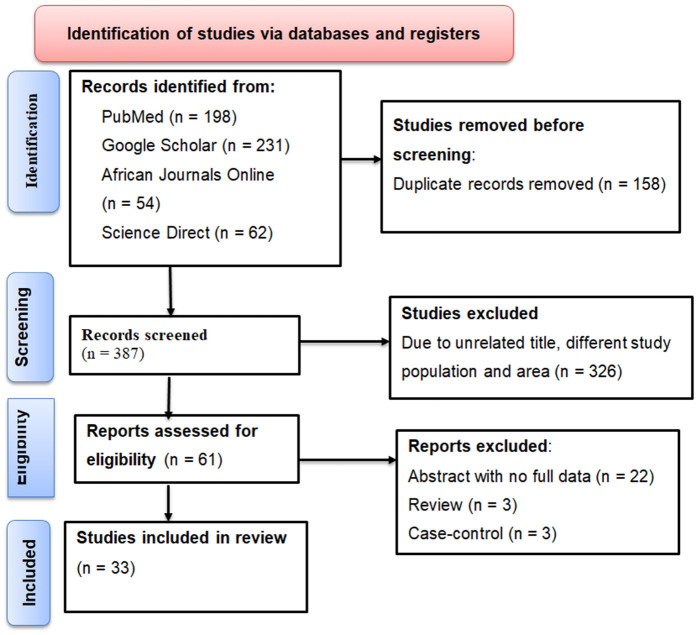
PRISMA study selection flow Diagram on the proportion of hookworm infection to intestinal helminths among pregnant women in Ethiopia.

An electronic database search initially identified 543 studies. After removing duplicates, 387 unique records remained. Of these, 326 articles were excluded after reviewing their titles and abstract for not meeting the inclusion criteria. Reasons for exclusion included studies not involving pregnant women, absence of data on hookworms prevalence, non-original study designs (e.g., reviews, editorials, case reports), and insufficient or incomplete data. Following the application of inclusion and exclusion criteria, a total of 33 studies were included for the proportion of infection with hookworms among intestinal helminths. The selection process is summarized in Fig 1.‌‌

### Characteristics of included articles

Thirty-one cross-sectional, and two prospective cohort studies were included. Regarding the study site (Community or facility base), 28 studies were facility base studies. Half (50%) of the studies were conducted in the Amhara region, 10 in the South nation, nationality of people (SNNP) region, five in the Oromia region, and one in each region of Tigray, Gambella, and Harerege. The sample size ranged from 180 [24] to 908 [25], and a total of 13,367 pregnant women were included in this final meta-analysis (Table 1).

**Table 1:** Summary characteristics of studies included in the systematic review and meta-analysis of the prevalence of infection with hookworms to intestinal helminths among pregnant women in Ethiopia, 2010–2024.

Author	Study setting	Study design	Region	Study period	Methods of DX	Sample size	Cases Hookworms	Prevalence (%)
Kumera G. *et al*., 2018 [26]	Institution based	cross-sectional	Amhara	July and August 2016	Direct wet-mount and Concentration	234	20	8.55 (5.33–12.67)
Feleke B.E. *et al*., 2018 [27]	Community based	cross-sectional	Amhara	November 2015 to January 2016	Concentration	783	111	14.18 (11.57–16.43)
Lebso M. *et al*., 2017 [28]	Community based	cross-sectional	SNNPR	May–June 2015	Direct wet-mount	507	54	10.65 (8.28–13.72)
Getachew M. *et al*., 2013 [29]	Community based	cross-sectional	SNNPR	August to September, 2011	Concentration	388	114	29.38 (24.48–33.52)
Shiferaw M.B. *et al*., 2015 [30]	institution-based	cross-sectional	Amhara	March to June 2015	Direct wet-mount	464	68	14.66 (11.75–18.25)
Tesfay D.J. *et al*., 2015 [31]	Institution based	cross-sectional	SNNPR	October 1–30, 2013	Direct wet-mount	258	18	6.98 (3.89–10.11)
Hailu T. *et al*., 2020 [32]	Institution based	cross-sectional	Amhara	February 2017 to June 2017	Concentration	743	138	18.57 (16.18–21.82)
Mengist H.M. *et al*., 2017 [24]	Institution based	cross-sectional	Oromia	November 2015 and January 2016	Direct wet-mount and Concentration	372	58	15.59 (12.27–19.73)
Alula G.A. *et al*., 2021 [33]	Institution based	cross-sectional	Amhara	November 2018 to March 2019	Direct wet-mount and Concentration	384	20	5.21 (2.82–18)
Belyhun Y. *et al*., 2010 [25]	Community based	Cohort	SNNPR	July 2006 and June 2007	Concentration	908	328	36.12 (32.88–39.12)
Tefera Girum, 2014 [34]	Institution based	Cross1-sectional	SNNPR	April, 1/2014–June 30/ 2014	Direct wet-mount and Concentration	748	128	17.11 (14.31–19.69)
Bolka M. *et al*., 2019 [35]	Institution based	cross-sectional	SNNPR	June and July 2018	Direct wet-mount and Concentration	352	39	11.08 (7.73–14.27)
Gebrehiwet M.G. *et al*., 2019 [36]	Institution based	cross-sectional	Amhara	2018	Concentration	448	179	39.96 (35.46–44.54)
Derso A. *et al*., 2016 [37]	Institution based	cross-sectional	Amhara	November 2013 to January 2014	Concentration	384	21	5.47 (2.82–7.18)
Aschale Y. *et al*., 2022 [38]	Community based	cross-sectional	Amhara	March 2021 to July 2021	Direct wet-mount	363	114	31.40 (26.24–35.76)
Kefyalew F. *et al*., 2014 [39]	Institution based	cross-sectional	Harerege	March to June, 2013	Concentration	258	16	6.20 (3.1–8.9)
Yesuf D.A. *et al*., 2019 [40]	Institution based	cross-sectional	Oromia	April 1 to May 15, 2019	Direct wet-mount and Concentration	315	106	33.65 (28.77–39.23)
Shiferaw M.B. *et al*., 2017 [41]	Institution based	cross-sectional	Amhara	March to June, 2015	Direct wet-mount	180	36	20.00 (14.16–25.84)
Damtie D. *et al*., 2021 [42]	Institution based	cross-sectional	Amhara	30 November 2019 to 07 March 2020	Direct wet-mount and Concentration	280	25	8.93 (5.65–12.35)
Demeke G. *et al.*, 2021 [43]	Institution based	Cohort	Amhara	December 2017 to February 2019.	concetration	278	48	17.27 (12.58–21.42)
Bekele A. *et al*., 2016 [44]	Institution based	cross-sectional	SNNPR	February 16 to April 8, 2015	Direct concentration	332	2	0.60 (−0.07 to 2.07)
Alemayehu A. *et al*., 2016 [45]	Institution based	cross-sectional	Gambela	April 15 to June 30, 2015	Direct wet-mount and concentration	360	16	4.44 (1.98–6.02)
Ejeta E. *et al*., 2014 [46]	Institution based	cross-sectional	Oromia	April to May, 2014	Direct wet-mount	286	11	3.85 (1.73–6.27)
Gedefaw L. *et al*., 2014 [47]	Institution based	cross-sectional	SNNPR	January to March 2014	Direct wet-mount	363	11	3.03 (1.25–4.75)
Kumera G. *et al*., 2018 [48]	Institution based	cross-sectional	Amhara	January and February 2016	Direct wet-mount and Concentration	409	13	3.18 (1.35–4.65)
Buchala A.D. *et al*., 2022 [49]	Institution based	cross-sectional	Southern Ethiopia	16 January to 15 February 2021	Direct wet-mount and Concentration	400	11	2.75 (1.33–4.67)
						286	39	13.64 (9.98–18.02)
Yesuf N.N. *et al*., 2021 [50]	Institution based	cross-sectional	Amhara	September to December 2019	Direct wet-mount	335	10	2.99 (1.17–4.83)
						377	19	5.04 (2.8–7.2)
Kebede E. *et al*., 2022 [51]	Institution based	cross-sectional	Amhara	October 2018 to February 2019	Direct wet-mount and concentration	365	17	4.66 (2.76–7.24)
Kumera G. *et al*., 2015 [52]	Institution based	cross-sectional	Amhara	March to May, 2014	Direct wet- mount	407	12	2.95 (1.34–4.66)
						384	18	4.69 (2.82–7.18)
Wachamo D. *et al*., 2021 [53]	Institution based	cross-sectional	Southern Ethiopia	September 01, 2019, up to Jan 30, 2020	Direct wet-mount and Concentration	416	33	7.93 (5.39–10.61)
Getachew M. *et al*., 2021 [54]	Institution based	cross-sectional	Oromia	March to June, 2016	concentration	234	20	8.55 (5.33–12.67)
Alem M. *et al*., 2013 [55]	Institution based	cross-sectional	Amhara	February to May 2011	Direct wet-mount	783	111	14.18 (11.57–16.43)
Mekonen AT, 2024 [56]	Institution based	cross-sectional	Oromia	May to June 2023	Direct concentration	507	54	10.65 (8.28–13.72)

### Prevalence

The prevalence of infection with hookworms varied considerably across different regions of Ethiopia. In the Amhara region, the reported prevalence ranged from 2.9% to 40.0%. In the Southern Nations, Nationalities and Peoples Region (SNNPR), the prevalence ranged from 0.6% to 36.1%. In the Oromia region, the prevalence ranged from 3.8% to 33.7%. In Gambela, the prevalence was 4.4%, while in Harerge it was 6.2%. Studies conducted in Southern Ethiopia reported prevalence values of 2.8% and 7.9% (Table 1).

### Proportion

From the total included articles, 33 studies reported a proportion of infection with hookworms to intestinal helminths. The proportion of infection with hookworms ranged from 10.2% [53] to 95.2% [32] (Table 2).‌‌

**Table 2:** Summary characteristics of studies included in the systematic review and meta-analysis of the proportion of infection with hookworms to intestinal helminths among pregnant women in Ethiopia, 2010–2024.

Author	Study setting	Study design	Region	Study period	Methods of DX	Number of intestinal helminthes	Cases Hookworm	Proportion 95% CI
Kumera G. *et al*., 2018 [26]	Institution based	cross-sectional	Amhara	July and August 2016	Direct wet-mount and Concentration	38	20	52.6 (36.8–68.5)
Feleke B.E. *et al*., 2018 [27]	Community based	cross-sectional	Amhara	November 2015 to January 2016	Concentration	595	111	18.7 (15.5–21.8)
Lebso M. *et al*., 2017 [28]	Community based	cross-sectional	SNNPR	May-June 2015	Direct wet-mount	161	54	33.5 (26.2–40.8)
Getachew M. *et al*., 2013 [29]	Community based	cross-sectional	SNNPR	August to September, 2011	Concentration	159	114	71.7 (64.7–78.7)
Shiferaw– M.B. *et al*., 2015 [30]	institution-based	cross-sectional	Amhara	March to June 2015	Direct wet-mount	97	68	70.1 (61–79.2)
Tesfay D.J. *et al*., 2015 [31]	Institution based	cross-sectional	SNNPR	October 1–30, 2013	Direct wet-mount	76	18	23.7 (14.1–33.2)
Hailu T. *et al*., 2020 [32]	Institution based	cross-sectional	Amhara	February 2017 to June 2017	Concentration	145	138	95.2 (91.7–98.7)
Mengist H.M. *et al*., 2017 [24]	Institution based	cross-sectional	Oromia	November 2015 and January 2016	Direct wet-mount and Concentration	92	58	63 (53.2–72.9)
Alula G.A. *et al*., 2021 [33]	Institution based	cross-sectional	Amhara	November 2018 to March 2019	Direct wet-mount and Concentration	76	20	26.3 (16.4–36.2)
Belyhun Y. *et al*., 2010 [25]	Community based	Cohort	SNNPR	July 2006 and June 2007	Concentration	395	328	83 (79.3–86.7)
Tefera Girum, 2014 [34]	Institution based	cross-sectional	SNNPR	April, 1/2014–June 30/ 2014	Direct wet-mount and Concentration	436	128	29.4 (25.1–33.6)
Bolka M. *et al*., 2019 [35]	Institution based	cross-sectional	SNNPR	June and July 2018	Direct wet-mount and Concentration	135	39	28.9 (21.2–36.5)
Gebrehiwet M.G. *et al*., 2019 [36]	Institution based	cross-sectional	Amhara	2018	Concentration	229	179	78.2 (72.8–83.5)
Derso A. *et al*., 2016 [37]	Institution based	cross-sectional	Amhara	November 2013 to January 2014	Concentration	66	21	31.8 (20.6–43.1)
Aschale Y. *et al*., 2022 [38]	Community based	cross-sectional	Amhara	March 2021 to July 2021	Direct wet-mount	120	114	95 (91.1–98.9)
Kefyalew F. *et al*., 2014 [39]	Institution based	cross-sectional	Harerege	March to June, 2013	Concentration	96	16	16.7 (9.2–24.1)
Yesuf D.A. *et al*., 2019 [40]	Institution based	cross-sectional	Oromia	April 1 to May 15, 2019	Direct wet-mount and Concentration	135	106	78.5 (71.6–85.4)
Shiferaw M.B. *et al*., 2017 [41]	Institution based	cross-sectional	Amhara	March to June, 2015	Direct wet-mount	38	36	94.7 (87.6–101.8)
Damtie D. *et al*., 2021 [42]	Institution based	cross-sectional	Amhara	30 November 2019 to 07 March 2020	Direct wet-mount and Concentration	99	25	25.3 (16.7–33.8)
Demeke G. *et al*., 2021 [43]	Institution based	Cohort	Amhara	December 2017 to February 2019.	concetration	74	48	64.9 (54–75.7)
Bekele A. *et al*., 2016 [44]	Institution based	cross-sectional	SNNPR	February 16 to April 8, 2015	Direct concentration	5	2	40 (−2.9 to 82.9)
Alemayehu A. *et al*., 2016 [45]	Institution based	cross-sectional	Gambela	April 15 to June 30, 2015	Direct wet-mount and concentration	68	16	23.5 (13.4–33.6)
Ejeta E. *et al*., 2014 [46]	Institution based	cross-sectional	Oromia	April to May, 2014	Direct wet-mount	22	11	50 (29.1–70.9)
Gedefaw L. *et al*., 2014 [47]	Institution based	cross-sectional	SNNPR	January to March 2014	Direct wet-mount	50	11	22 (10.5–33.5)
Kumera G. *et al*., 2018 [48]	Institution based	cross-sectional	Amhara	January and February 2016	Direct wet-mount and Concentration	74	13	17.6 (8.9–26.2)
Buchala A.D. *et al*., 2022 [49]	Institution based	cross-sectional	Southern Ethiopia	16 January to 15 February 2021	Direct wet-mount and Concentration	67	11	16.4 (7.5–25.3)
Yesuf N.N. *et al*., 2021 [50]	Institution based	cross-sectional	Amhara	September to December 2019	Direct wet-mount	57	39	68.4 (56.4–80.5)
Kebede E. *et al*., 2022 [51]	Institution based	cross-sectional	Amhara	October 2018 to February 2019	Direct wet-mount and concentration	72	10	13.9 (5.9–21.9)
Kumera G. *et al*., 2015 [52]	Institution based	cross-sectional	Amhara	March to May, 2014	Direct wet- mount	57	19	33.3 (21.1–45.6)
Wachamo D. *et al*., 2021 [53]	Institution based	cross-sectional	Southern Ethiopia	September 01, 2019, upto January 30, 2020	Direct wet-mount and Concentration	167	17	10.2 (5.6–14.8)
Getachew M. *et al*., 2021 [54]	Institution based	cross-sectional	Oromia	March to June, 2016	concentration	80	12	15 (7.2–22.8)
Alem M. *et al*., 2013 [55]	Institution based	cross-sectional	Amhara	February to May 2011	Direct wet-mount	55	18	32.7 (20.3–45.1)
Mekonen AT, 2024 [56]	Institution based	cross-sectional	Oromia	May to June 2023	Direct concentration	125	33	26.4 (18.7–34.1)

Dx, diagnosis.

### Meta-analysis

#### Prevalence.

**Fig 2:**
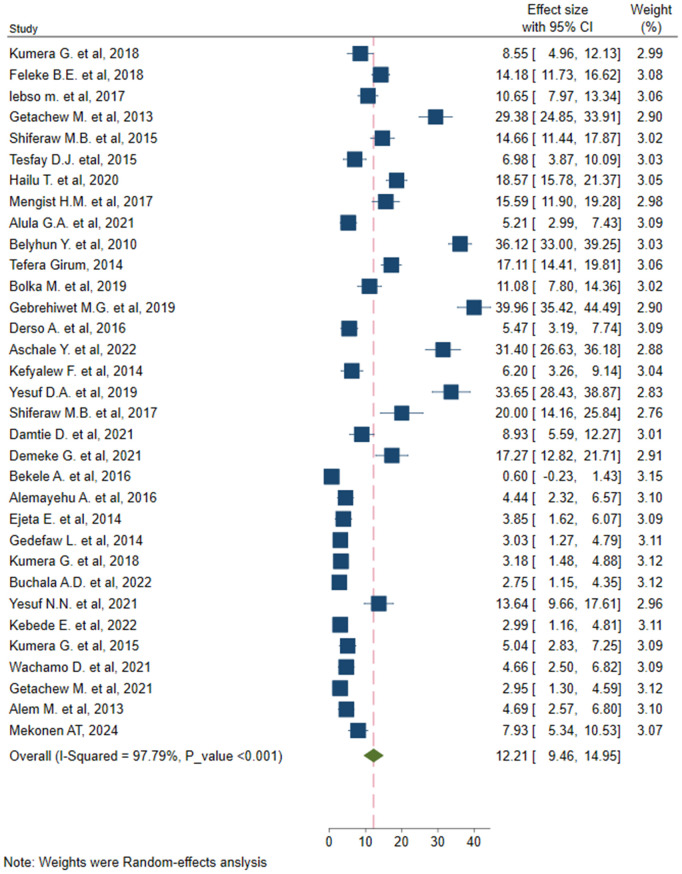
Forest plot of the prevalence of hookworm infection among pregnant women in Ethiopia, 2010–2024.

**Fig 3:**
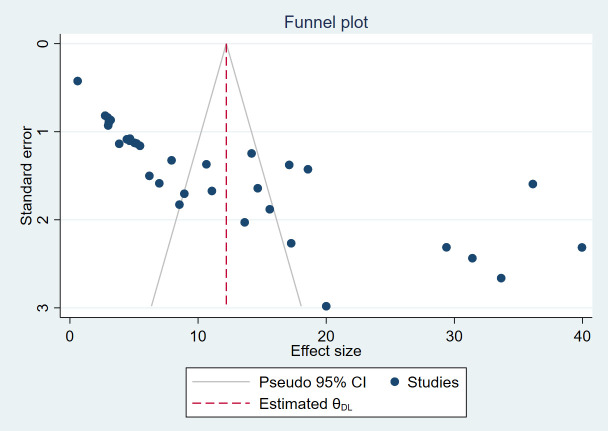
Funnel plot assessing publication bias in the prevalence of hookworm infection among pregnant women in Ethiopia, 2010–2024.

The pooled prevalence of infection with hookworms among pregnant women in Ethiopia was 12.21% (95% CI: 9.46–14.95). There was considerable heterogeneity among the included studies (I² = 97.79%, *p* < 0.001) (Fig 2). Egger’s test suggested possible publication bias (*p* < 0.001); however, trim-and-fill analysis did not impute additional studies and the pooled estimate remained unchanged, indicating a negligible effect of publication bias (Fig 3).

#### Proportion of hookworm.

**Fig 4:**
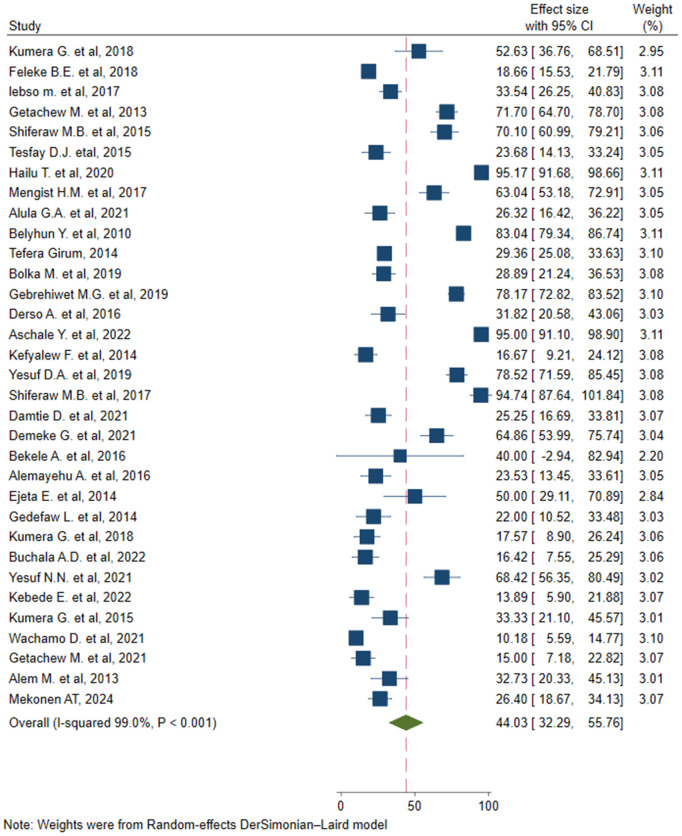
Forest plot of the proportion of hookworm infection among pregnant women in Ethiopia, 2010–2024.

**Fig 5:**
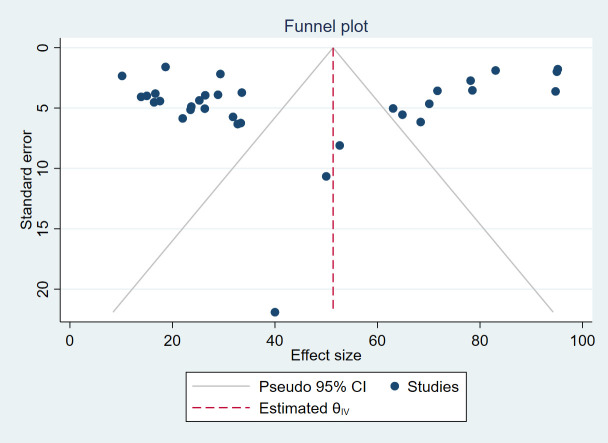
Funnel plot for assessing the presence of publication bias in studies reporting the proportion of hookworm infection among pregnant women with intestinal helminth infections in Ethiopia, 2010–2024.

The pooled proportion of infection with hookworms to intestinal helminths was 44.03% (95% CI: 32.29%–55.76%). Significant heterogeneity was observed between studies (I^2^ = 99.0%, *P* < 0.001) (Fig 4). The funnel plot (Fig 5) and the asymmetry Egger’s test [b: −0.94] (95% CI; −4.61 to 2.73, *P* = 0.6293) did not show evidence of publication bias.

#### Sensitivity analysis results.

**Fig 6:**
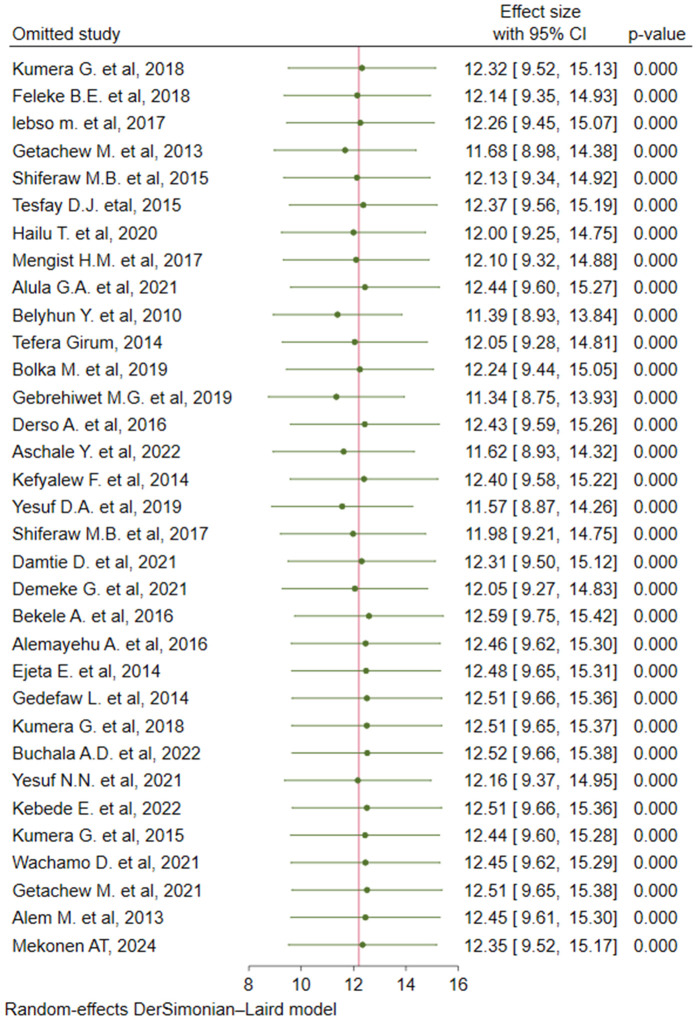
A forest plot depicting leave-one-out sensitivity analysis to estimate the pooled prevalence of hookworm infection among pregnant women in Ethiopia, 2010–2024.

The leave-one-out sensitivity analysis, conducted using a random-effects (DerSimonian–Laird) model, assessed the influence of each individual study on the overall pooled prevalence of infection with hookworms. The analysis demonstrated that the pooled estimate was robust and stable. When each study was omitted in turn, the resulting pooled effect sizes remained tightly clustered, ranging from ~11.34% to 12.59%. The 95% CIs for these estimates showed substantial overlap, consistently spanning from the low 8% to the mid-15% range. Notably, no single omission caused the pooled estimate to fall outside the CIs of the overall analysis, indicating that no individual study exerted a disproportionate influence on the meta-analysis findings (Fig 6).

**Fig 7:**
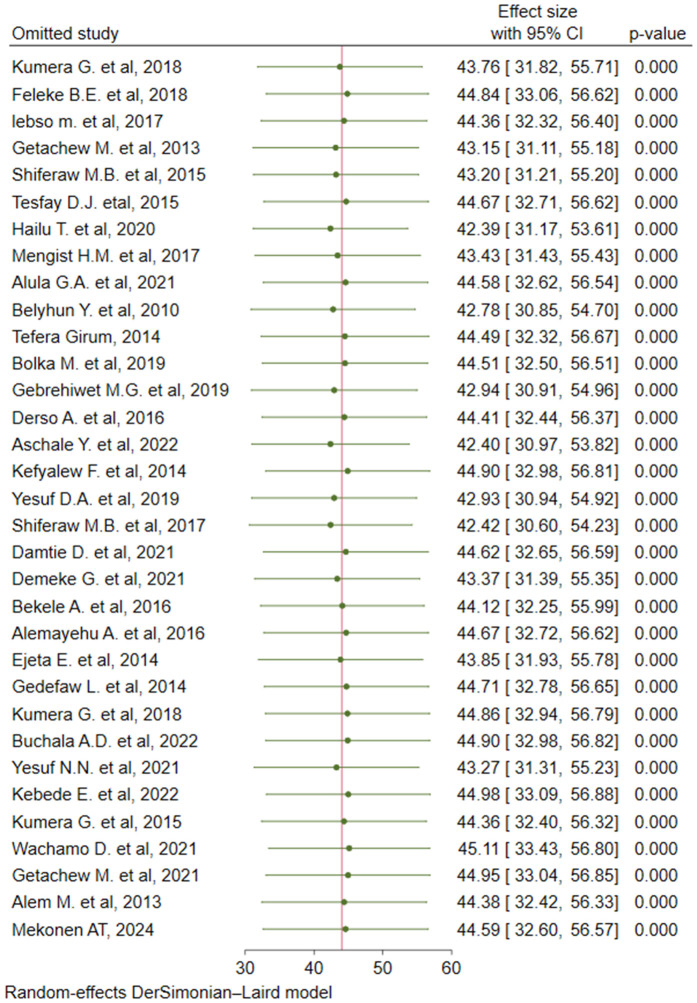
A forest plot showing leave-one-out sensitivity analysis for the pooled proportion of hookworm infection among pregnant women infected with intestinal helminthes.

A leave-one-out sensitivity analysis was performed to assess the influence of each individual study on the overall pooled estimate of proportion of infection with hookworms. The analysis demonstrated that the pooled proportion remained stable and robust, with the overall effect size remaining consistently within a narrow range (~42% to 45%) upon the exclusion of any single study. The 95% CIs for these pooled estimates also remained stable, indicating minimal fluctuation in precision. No individual study was found to disproportionately influence the global estimate, as the point estimate from the meta-analysis remained well within the CIs of the overall effect when each study was omitted in turn. (Fig 7).

#### Subgroup analysis.

**Fig 8:**
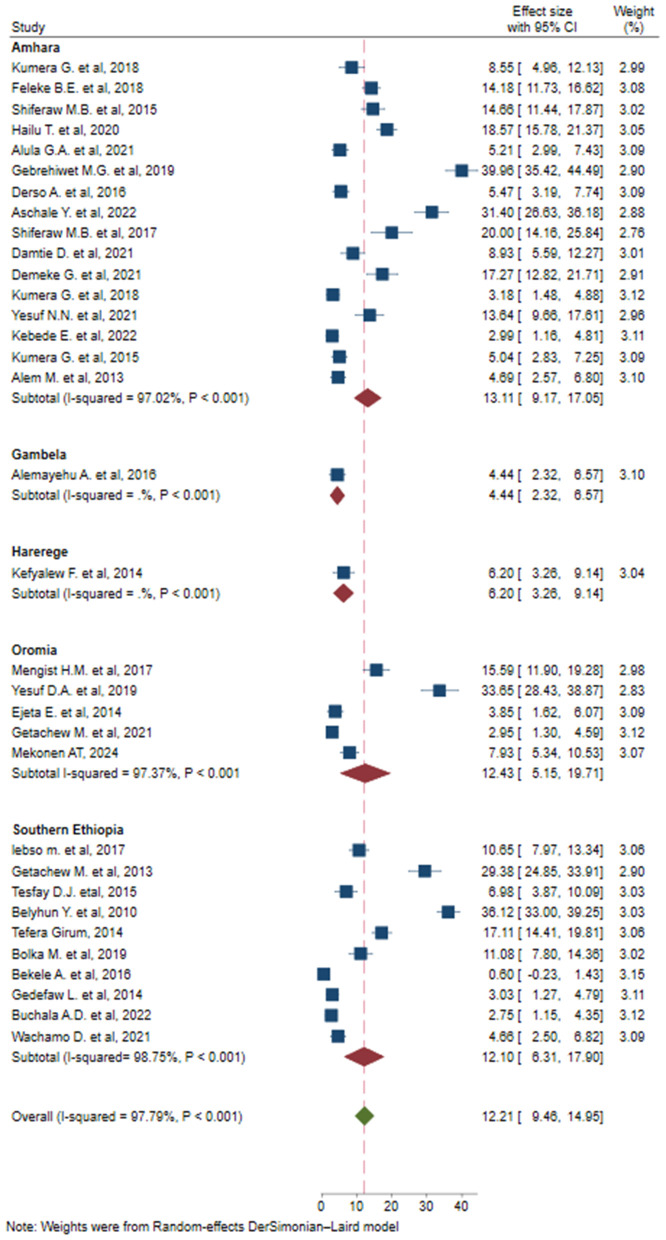
Forest plot of regional subgroup analysis of the prevalence of hookworm infection among pregnant women in Ethiopia, 2010–2024.

The forest plot shows the subgroup analysis of infection with hookworms among pregnant women with intestinal helminth infections in Ethiopia using a random-effects (DerSimonian–Laird) model. The prevalence varied across regions, with the highest prevalence observed in Amhara (13.11%, 95% CI: 9.17%–17.05%) and the lowest in Gambela (4.44%, 95% CI: 2.32%–6.57%) (Fig 8).

**Fig 9:**
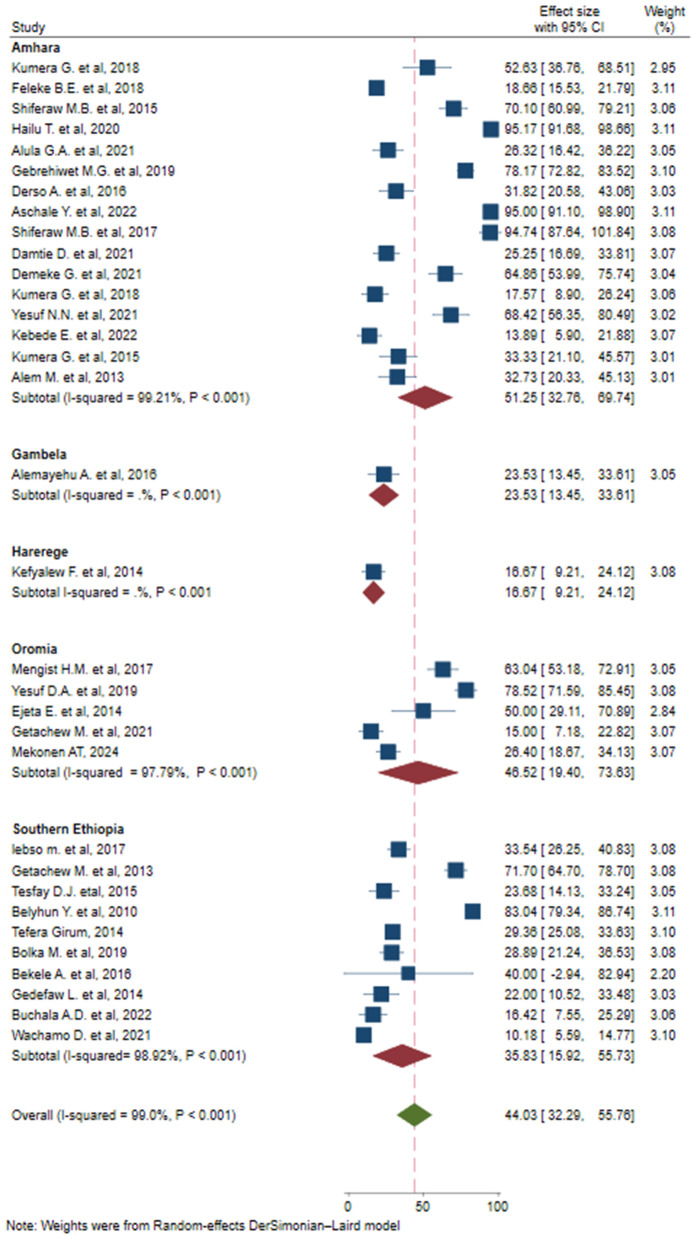
Forest plot of regional subgroup analysis of the proportion of hookworm infection among pregnant women with intestinal helminth infections in Ethiopia, 2010–2024.

The subgroup analysis across regions revealed considerable variation in the pooled proportion of infection with hookworms in Ethiopia. The highest pooled proportion of infection with hookworms was observed in the Amhara region at 51.25% (95% CI: 32.76%–69.74%), while the lowest was observed in the Harerege region at 16.67% (95% CI: 9.21%–24.12%) (Fig 9).

**Fig 10:**
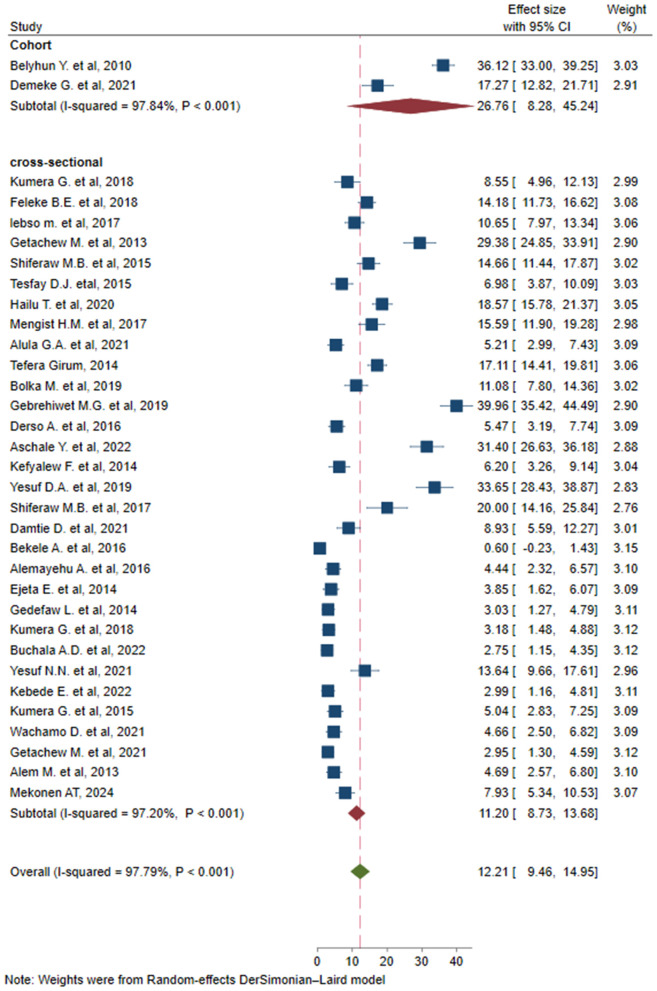
Forest plot of study design subgroup analysis of the prevalence of hookworm infection among pregnant women in Ethiopia, 2010–2024.

**Fig 11:**
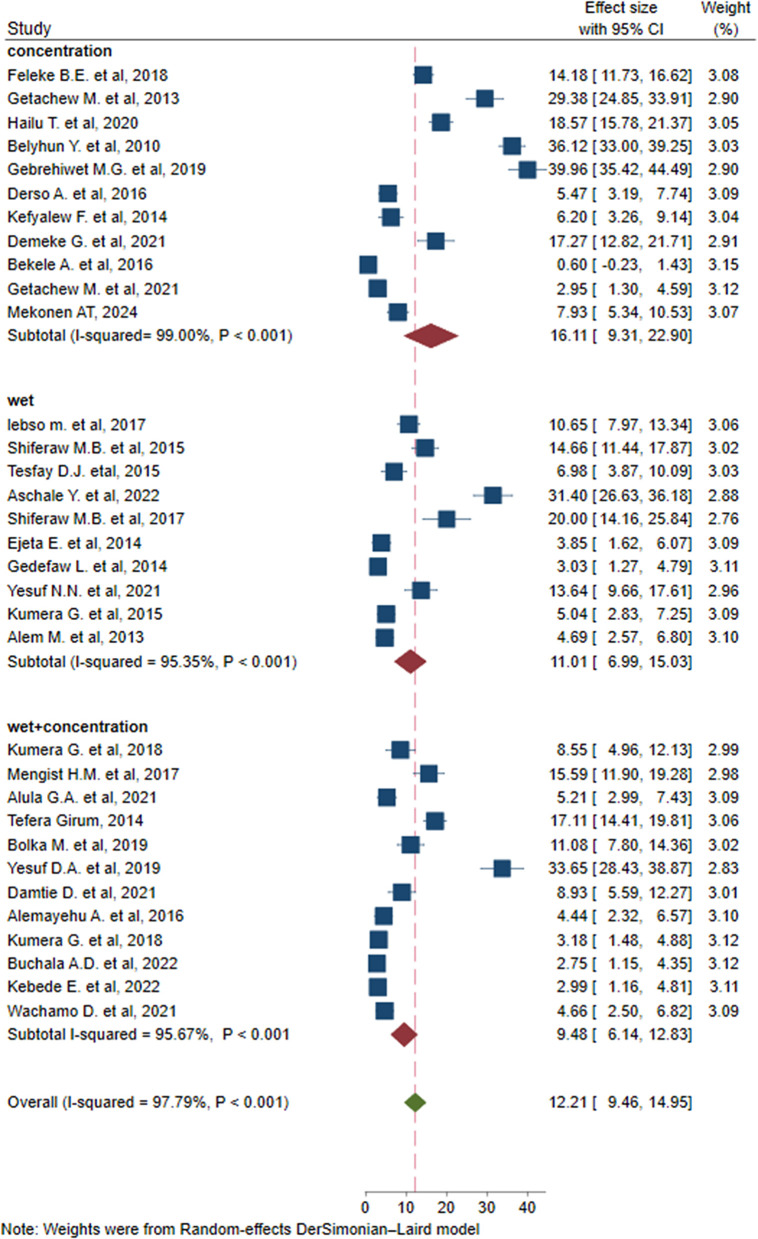
Forest plot of diagnostic method subgroup analysis of the prevalence of hookworm infection among pregnant women in Ethiopia, 2010–2024.

**Fig 12:**
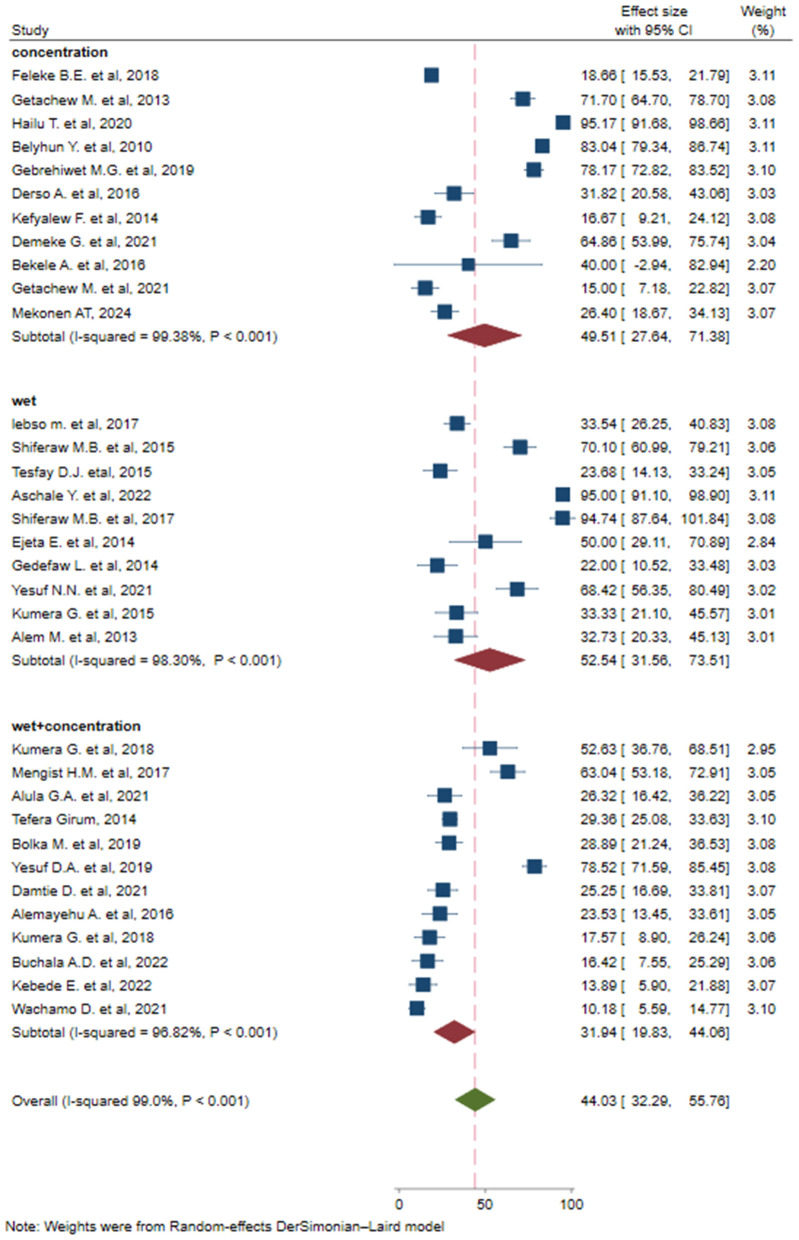
Forest plot of methods of diagnosis subgroup analysis of the proportion of hookworm infection among pregnant women with intestinal helminth infections in Ethiopia, 2010–2024.

The subgroup analysis revealed a notable discrepancy in the pooled estimates depending on study design. The two cohort studies included in the analysis yield a pooled prevalence of 26.76% (95% CI: 8.28%–45.24%). In contrast, the 31 cross-sectional studies provided a much more precise pooled prevalence of 11.20% (95% CI: 8.73%–13.68%) (Fig 10). Subgroup analysis by diagnostic method showed that the pooled prevalence of infection with hookworms varied, with the highest estimate from studies using the concentration method at 16.11% (95% CI: 9.31%–22.90%), and the lowest from combined wet-mount and concentration methods at 9.48% (95% CI: 6.14%–12.83%) (Fig 11). The forest plot presents a subgroup meta-analysis of infection with hookworms proportion among pregnant women based on diagnosis methods categorized as concentration, wet, and wet plus concentration methods. The highest pooled proportion was observed in wet areas at 52.54% (95% CI: 31.56%–73.51%), while the lowest was in wet plus concentration method at 31.94% (95% CI: 19.83%–44.08%). The concentration method had a pooled proportion of 49.51% (95% CI: 27.64%–71.38%) (Fig 12).

**Fig 13:**
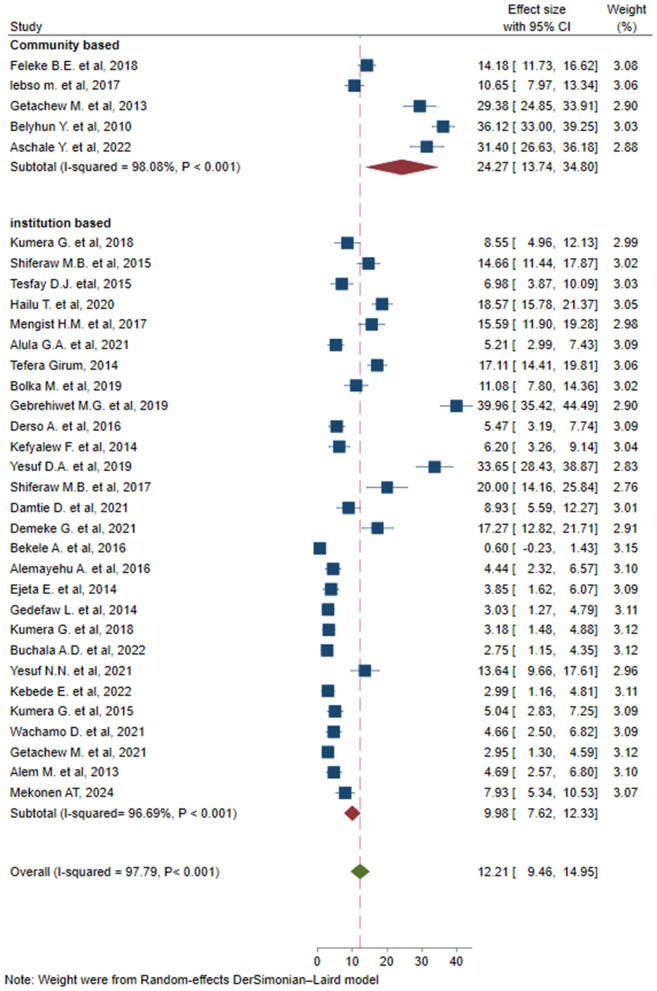
Forest Plot of subgroup analysis by study setting of the prevalence of hookworm infection among pregnant women in Ethiopia, 2010–2024.

The forest plot shows that the subgroup analysis by study setting, community based studies reported a notably higher pooled prevalence of 24.27% (95% CI: 13.74%–34.80%), whereas institution-based studies showed a lower prevalence of 9.98% (95% CI: 7.62%–12.33%) (Fig 13).

**Fig 14:**
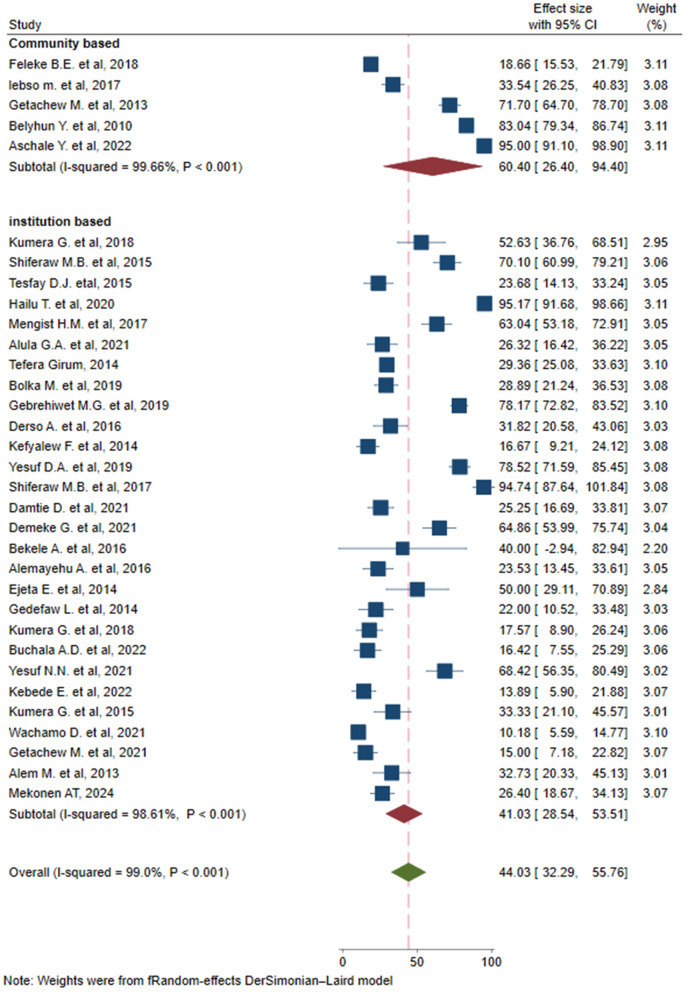
Forest Plot of subgroup analysis by study setting of the proportion of hookworm infection among pregnant women in Ethiopia, 2010–2024.

Subgroup analysis by study setting revealed that community based studies had a higher pooled proportion of 60.40% (95% CI: 26.40%–94.40%), while institution-based studies reported a lower-proportion of 41.03% (95% CI: 28.54%–53.51%) (Fig 14).

### Meta-regression

A multivariable random-effects meta-regression using the restricted maximum likelihood (REML) method was conducted to explore potential sources of heterogeneity among the 33 included studies. The model included sample size, publication year, region, study design, diagnostic technique, and study setting as moderators. The overall model was not statistically significant (Wald χ² [10] = 11.88, *p* = 0.293), indicating that these variables collectively did not significantly explain the variability in effect sizes across studies. The included moderators explained only 5.66% of the between-study heterogeneity (R² = 5.66%), while substantial heterogeneity remained (τ² = 104.2; I² = 98.41%), suggesting considerable variability among the included studies.

Regarding individual covariates, sample size (β = 0.003, *p* = 0.844) and publication year (β = −0.334, *p* = 0.635) were not significantly associated with the pooled effect size. Similarly, geographic region (Gambela, Harerege, Oromia, and Southern Ethiopia), study design (cross-sectional), and diagnostic techniques (wet-mount and wet plus concentration methods) did not show statistically significant associations with the prevalence of infection with hoockworms (all *p* > 0.05). However, the study setting was significantly associated with the effect size. Institution-based studies reported significantly lower prevalence compared with the reference category (β = −12.88, *p* = 0.040). The test of residual heterogeneity remained statistically significant (Q_res = 702.15, *p* < 0.001), indicating that important sources of heterogeneity were still unexplained by the included moderators (Table 3).

**Table 3:** Meta-regression analysis of factors associated with the prevalence of infection with hookworms variation across studies in Ethiopia, 2010–2024.

Variable	Coefficient (β)	Std. Error	*z*-value	*p*-value	95% CI
Sample size	0.003	0.015	0.20	0.844	−0.026 to 0.032
Published year	−0.334	0.704	−0.47	0.635	−1.713 to 1.045
Region					
Gambela	−8.31	11.33	−0.73	0.463	−30.53 to 13.90
Harerege	−6.93	12.35	−0.56	0.575	−31.14 to 17.27
Oromia	1.32	5.44	0.24	0.808	−9.35 to 11.99
Southern Ethiopia	−4.80	4.61	−1.04	0.298	−13.83 to 4.23
Study design					
Cross-sectional	−9.07	8.54	−1.06	0.288	−25.80 to 7.67
Diagnostic technique					
Wet-mount	−2.60	5.72	−0.46	0.649	−13.81 to 8.60
Wet + concentration	−0.01	5.19	−0.00	0.999	−10.18 to 10.16
Study setting					
Institution-based	−12.88	6.27	−2.05	0.040	−25.17 to −0.58
Constant	706.44	1421.72	0.50	0.619	−2080.07 to 3492.96

The random-effects meta-regression was conducted to explore whether study characteristics explain the heterogeneity among the 33 included studies. The model included variables such as sample size, publication year, region, study design, diagnostic technique, and study setting.

The results showed that none of the covariates were statistically significant predictors of the pooled effect size. Specifically, sample size (β = −0.026, *p* = 0.694) and publication year (β = −2.25, *p* = 0.409) were not significantly associated with the outcome. Similarly, regional categories (Gambela, Hararghe, Oromia, and Southern Ethiopia), study design (cross-sectional), diagnostic techniques (wet-mount and wet plus concentration methods), and study setting (institution-based) did not significantly influence the pooled proportion of infection hoockworms, as all *p*-values were greater than 0.05.

Furthermore, the model indicated no remaining heterogeneity, with a between-study variance of τ² = 0 and I² = 0%, suggesting that the included covariates explained nearly all variability across studies. However, the overall model was not statistically significant (Wald χ² [10] = 3.60, *p* = 0.9635), indicating that the selected variables collectively did not significantly explain variations in effect sizes. In addition, the test of residual homogeneity (Q_res = 5.70, *p* = 0.9998) suggests that there was no significant unexplained heterogeneity remaining after the meta-regression model (Table 4).

**Table 4:** Meta-regression analysis of factors associated with proportion of infection with hookworms variation across studies in Ethiopia, 2010–2024.

Covariate	Coefficient (β)	Standard Error	*z*-value	*p*-value	95% Confidence Interval
Sample size	−0.026	0.066	−0.39	0.694	−0.155, 0.103
Study year	−2.253	2.729	−0.83	0.409	−7.601, 3.095
Region					
Gambela	−7.739	27.968	−0.28	0.782	−62.556, 47.077
Hararghe	−21.355	31.081	−0.69	0.492	−82.272, 39.562
Oromia	4.163	21.532	0.19	0.847	−38.038, 46.365
Southern Ethiopia	−6.100	10.437	−0.58	0.559	−26.557, 14.356
Design					
Cross-sectional	−45.632	53.176	−0.86	0.391	−149.855, 58.592
Technique					
Wet	2.184	23.942	0.09	0.927	−44.742, 49.110
Wet + Concentration	0.396	19.612	0.02	0.984	−38.043, 38.834
Setting					
Institution-based	−9.218	28.493	−0.32	0.746	−65.063, 46.628
Constant (_cons)	4636.567	5525.644	0.84	0.401	−6193.497, 15466.63

## Discussion

The findings of this study indicated that infection with hookworms among pregnant women in Ethiopia remains a significant public health concern, with prevalence varying considerably across regions. The highest prevalence was reported in the Amhara region, ranging from 2.9% to 40.0%, while Gambela and Harerge showed much lower prevalence rates of 4.4% and 6.2%, respectively. These regional differences may reflect variations in environmental conditions, sanitation infrastructure, socioeconomic status, and access to preventive interventions such as deworming programs during pregnancy. The pooled prevalence of infection hookworms among pregnant women in Ethiopia was 12.21% (95% CI: 9.46%–14.95%), highlighting a moderate overall burden of infection in this population. The high heterogeneity observed across studies (I² = 97.79%, *p* < 0.001) suggests substantial variability in study populations, diagnostic methods, and sampling periods, which should be considered when interpreting the results. Although Egger’s test indicated potential publication bias (*p* < 0.001), the trim-and-fill analysis did not adjust the pooled estimate, suggesting that any bias had a negligible effect on the overall prevalence estimate.

This systematic review and meta-analysis also provide complete evidence for the proportion of infection with hookworms in pregnant women in Ethiopia, highlighting its continued public health significance [57,58]. As much as with a relatively higher proportion in the pooled estimation relative to reporting across the globe, the disease remains very much prevalent in specific locations and constitutes a vast majority of intestinal helminthic infections in the target population. The study found that pregnant women had a pooled proportion of infection with hookworms of 44.0% (95% CI: 33.0%–55.0%), higher than the prevalence of infection hookworms in the general population, which varied from 10% to 30% in Ethiopia [59,60]. This might be due to pregnant women are often exposed to contaminants in the soil through agricultural activity or barefoot walking in many areas with limited resources, which raises their risk of infection [61,62]. The risk is also increased by their lack of access to prenatal care and exclusion from mass deworming activities. Social and economic factors are also important; these include inadequate sanitation, low educational attainment, and restricted use of health services. The greater percentage of infection with hookworms seen in pregnant women compared to the general population can be explained by these interrelated facts [5].

The regional variations observed for proportion imply a multifactorial interplay of geographic, environmental, and socioeconomic determinants [5]. Tigray, for example, had a significantly higher proportion, likely due to differences in sanitation facility infrastructure, availability of clean water, practices of barefoot walking, health education, and coverage of mass deworming campaigns [63]. Alternatively, lower-proportion regions could be supported by enhanced health service coverage or continued control interventions, though the limited number of studies in some regions requires cautious interpretation.

The larger proportion in community based as compared to health facility-based studies implies that routine facility data are an underestimation of the true community-level proportion. Similarly, heterogeneity by study design, such as the larger estimates in cohort studies, can be explained by the advantages of longitudinal follow-up in capturing incident cases or adjusting for seasonality in transmission [64].

The high heterogeneity between studies, while to be expected in a national survey that utilizes different settings and methodologies, says a great deal regarding the need for more standardized study protocols and diagnostics [65,66]. Although the suggestion of publication bias in the analysis of proportion might affect the accuracy of the pooled estimate, the absence of publication bias in the analysis of proportions lends assurance to the integrity of hookworm’s dominance of intestinal helminths.

The findings also show the need to accord high priority to pregnant women in soil-transmitted helminths control. As the morbidity effects of infection with hookworms during pregnancy include maternal anemia, low birth weight, and poor neonatal outcomes, interventions like frequent deworming as part of antenatal care, improved sanitation, frequent shoe-wearing behavior, and community health education should be ramped up, especially in highly burdened communities [67].

### Strengths and limitations

This study has several strengths, including a comprehensive and systematic literature search that ensured broad coverage of relevant studies in Ethiopia, and the use of robust statistical methods such as random-effects models and subgroup analyses to provide reliable pooled estimates while accounting for variability. It also explored multiple study-level factors, offering deeper insight into sources of heterogeneity, and assessed both the prevalence and proportion of infection with hookworms for a more complete understanding of the burden. However, some limitations should be noted. Substantial heterogeneity across studies may affect the precision of the estimates, and variations in diagnostic methods, study designs, and sampling techniques could have influenced the results. The uneven geographic representation of studies limits generalizability, and potential publication bias cannot be ruled out. Additionally, the exclusion of unpublished or nonindexed studies may have led to missing relevant data, while the predominance of cross-sectional studies limits causal inference and the assessment of temporal trends.

## Conclusion

This meta-analysis demonstrated that infection with hookworms remains a significant public health concern among pregnant women in Ethiopia. Recent evidence suggests that hookworms’ infections remain relatively common despite ongoing control efforts. The pooled prevalence of infection with hookworms was 12.21%, indicating that a persistent burden of infection in this vulnerable population, although prevalence does not necessarily reflect the burden of clinical disease, which is more closely associated with moderate- to heavy-intensity infections. Furthermore, hookworms accounted for a substantial proportion (44.03%) of intestinal helminth infections, highlighting its dominance among helminthic infections in the study setting.

Notable regional variations were observed, with the highest prevalence reported in the Amhara region and the lowest in Gambella. Similarly, the proportion of hookworms among intestinal helminths varied widely, with the highest in Amhara and the lowest in Harerge.

Differences in diagnostic methods also affected the estimated prevalence, with concentration techniques yielding higher estimates compared to combined methods, underscoring the importance of sensitive diagnostic approaches in accurately determining disease burden.

Overall, the findings emphasize the need for targeted and region-specific interventions, including improved sanitation, health education, and routine deworming programs, particularly in high-burden areas. Strengthening diagnostic capacity and standardizing methodologies across studies are also essential to improve the accuracy and comparability of future research.

## Supporting information

S1 PRISMA Checklist PRISMA 2020 Checklist for prevalence of hookworm infection and its proportion among pregnant women in Ethiopia, 2010–2024. From: Page MJ, McKenzie JE, Bossuyt PM, Boutron I, Hoffmann TC, Mulrow CD, *et al*. The PRISMA 2020 statement: an updated guideline for reporting systematic reviews. *BMJ* 2021;372:n71. https://doi.org/10.1136/bmj.n71. This work is licensed under CC BY 4.0. To view a copy of this license, visit https://creativecommons.org/licenses/by/4.0/.
(DOCX)

S1 Data Data extraction form for prevalence of hookworm infection and its proportion among pregnant women in Ethiopia, 2010–2024. (XLSX)

S1 File Quality assessment results for prevalence of hookworm infection and its proportion among pregnant women in Ethiopia, 2010–2024. The data sets generated during the current study.
(DOCX)
