# Supplementary material for: Prevalence of hookworm infection and its proportion among pregnant women with intestinal helminth infection in Ethiopia: A systematic review and meta-analysis
Source: PLoS Negl Trop Dis. 2026 Jun 10;20(6):e0014376. doi: 10.1371/journal.pntd.0014376 (PMC13252847; doi:10.1371/journal.pntd.0014376)
Supplement: S1 File — The data sets generated during the current study. (DOCX) [file pntd.0014376.s003.docx]

Tables

| S.N | Name of Author | Published year | study setting | study design | Region | study period | Sample size | Technique of hookworm diagnosis | Number of cases for intestinal helminth | Number of Cases for Hookworm |
| --- | --- | --- | --- | --- | --- | --- | --- | --- | --- | --- |
| 1 |  |  |  |  |  |  |  |  |  |  |
| 2 |  |  |  |  |  |  |  |  |  |  |
| 3 |  |  |  |  |  |  |  |  |  |  |
| 4 |  |  |  |  |  |  |  |  |  |  |
| 5 |  |  |  |  |  |  |  |  |  |  |
| 6 |  |  |  |  |  |  |  |  |  |  |
| 7 |  |  |  |  |  |  |  |  |  |  |
| 8 |  |  |  |  |  |  |  |  |  |  |
| 9 |  |  |  |  |  |  |  |  |  |  |
| 10 |  |  |  |  |  |  |  |  |  |  |
| 11 |  |  |  |  |  |  |  |  |  |  |
| 12 |  |  |  |  |  |  |  |  |  |  |
| 13 |  |  |  |  |  |  |  |  |  |  |
| 14 |  |  |  |  |  |  |  |  |  |  |
| 15 |  |  |  |  |  |  |  |  |  |  |
| 16 |  |  |  |  |  |  |  |  |  |  |
| 17 |  |  |  |  |  |  |  |  |  |  |
| 18 |  |  |  |  |  |  |  |  |  |  |
| 19 |  |  |  |  |  |  |  |  |  |  |
| 20 |  |  |  |  |  |  |  |  |  |  |
| 21 |  |  |  |  |  |  |  |  |  |  |
| 22 |  |  |  |  |  |  |  |  |  |  |
| 23 |  |  |  |  |  |  |  |  |  |  |
| 24 |  |  |  |  |  |  |  |  |  |  |
| 25 |  |  |  |  |  |  |  |  |  |  |
| 26 |  |  |  |  |  |  |  |  |  |  |
| 27 |  |  |  |  |  |  |  |  |  |  |
| 28 |  |  |  |  |  |  |  |  |  |  |
| 29 |  |  |  |  |  |  |  |  |  |  |
| 30 |  |  |  |  |  |  |  |  |  |  |
| 31 |  |  |  |  |  |  |  |  |  |  |
| 32 |  |  |  |  |  |  |  |  |  |  |
| 33 |  |  |  |  |  |  |  |  |  |  |

| **Author (Year)** | **Selection (5)** | **Comparability (2)** | **Outcome (3)** | **Total Score** | **Quality** |
| --- | --- | --- | --- | --- | --- |
| Kumera G. et al (2018) | 4 | 1 | 2 | 7 | Moderate |
| Feleke B.E. et al (2018) | 5 | 1 | 2 | 8 | High |
| Lebso M. et al (2017) | 4 | 1 | 2 | 7 | Moderate |
| Getachew M. et al (2013) | 4 | 1 | 2 | 7 | Moderate |
| Shiferaw M.B. et al (2015) | 4 | 1 | 2 | 7 | Moderate |
| Tesfay D.J. et al (2015) | 3 | 1 | 2 | 6 | Moderate |
| Hailu T. et al (2020) | 5 | 1 | 2 | 8 | High |
| Mengist H.M. et al (2017) | 4 | 1 | 2 | 7 | Moderate |
| Alula G.A. et al (2021) | 4 | 1 | 2 | 7 | Moderate |
| Belyhun Y. et al (2010) | 5 | 2 | 3 | 10 | High |
| Tefera Girum (2014) | 4 | 1 | 2 | 7 | Moderate |
| Bolka M. et al (2019) | 4 | 1 | 2 | 7 | Moderate |
| Gebrehiwet M.G. et al (2019) | 5 | 1 | 2 | 8 | High |
| Derso A. et al (2016) | 4 | 1 | 2 | 7 | Moderate |
| Aschale Y. et al (2022) | 5 | 1 | 2 | 8 | High |
| Kefyalew F. et al (2014) | 3 | 1 | 2 | 6 | Moderate |
| Yesuf D.A. et al (2019) | 4 | 1 | 2 | 7 | Moderate |
| Shiferaw M.B. et al (2017) | 3 | 1 | 2 | 6 | Moderate |
| Damtie D. et al (2021) | 4 | 1 | 2 | 7 | Moderate |
| Demeke G. et al (2021) | 4 | 2 | 2 | 8 | High |
| Bekele A. et al (2016) | 3 | 1 | 2 | 6 | Moderate |
| Alemayehu A. et al (2016) | 4 | 1 | 2 | 7 | Moderate |
| Ejeta E. et al (2014) | 3 | 1 | 2 | 6 | Moderate |
| Gedefaw L. et al (2015) | 3 | 1 | 2 | 6 | Moderate |
| Kumera G. et al (2018) | 4 | 1 | 2 | 7 | Moderate |
| Buchala A.D. et al (2022) | 4 | 1 | 2 | 7 | Moderate |
| Yesuf N.N. et al (2021) | 4 | 1 | 2 | 7 | Moderate |
| Kebede E. et al (2022) | 4 | 1 | 2 | 7 | Moderate |
| Kumera G. et al (2015) | 3 | 1 | 2 | 6 | Moderate |
| Wachamo D. et al (2021) | 4 | 1 | 2 | 7 | Moderate |
| Getachew M. et al (2021) | 4 | 1 | 2 | 7 | Moderate |
| Alem M. et al (2013) | 3 | 1 | 2 | 6 | Moderate |
| Mekonen A.T (2024) | 5 | 1 | 2 | 8 | High |
